# Post-Translational Decrease in Respiratory Chain Proteins in the Polg Mutator Mouse Brain

**DOI:** 10.1371/journal.pone.0094646

**Published:** 2014-04-10

**Authors:** David N. Hauser, Allissa A. Dillman, Jinhui Ding, Yan Li, Mark R. Cookson

**Affiliations:** 1 Cell Biology and Gene Expression Section, Laboratory of Neurogenetics, National Institute on Aging, National Institutes of Health, Bethesda, Maryland, United States of America; 2 Brown University/National Institutes of Health Graduate Partnership Program, Department of Neuroscience, Brown University, Providence, Rhode Island, United States of America; 3 Department of Neuroscience, Karolinska Institutet, Stockholm, Sweden; 4 Computational Biology Unit, Laboratory of Neurogenetics, National Institute on Aging, National Institutes of Health, Bethesda, Maryland, United States of America; 5 Protein/Peptide Sequencing Facility, National Institute of Neurological Disorders and Stroke, National Institutes of Health, Bethesda, Maryland, United States of America; RIKEN Brain Science Institution, Japan

## Abstract

Mitochondrial DNA damage is thought to be a causal contributor to aging as mice with inactivating mutations in polymerase gamma (Polg) develop a progeroid phenotype. To further understand the molecular mechanisms underlying this phenotype, we used iTRAQ and RNA-Seq to determine differences in protein and mRNA abundance respectively in the brains of one year old Polg mutator mice compared to control animals. We found that mitochondrial respiratory chain proteins are specifically decreased in abundance in the brains of the mutator mice, including several nuclear encoded mitochondrial components. However, we found no evidence that the changes we observed in protein levels were the result of decreases in mRNA expression. These results show that there are post-translational effects associated with mutations in Polg.

## Introduction

Damage to mitochondrial DNA (mtDNA) is thought to be central to the process of aging and some age-related diseases [1], [2]. To monitor the effects of increased mtDNA damage *in vivo*, two groups independently generated lines of knock-in mice expressing a proofreading deficient version of the mtDNA polymerase gamma (Polg) in place of the wild type enzyme [3], [4]. Mice homozygous for the mutant allele (Polg ^D257A/D257A^, hereafter termed Polg mutator mice) exhibit a progeroid phenotype with aging phenotypes that include kyphosis, loss of subcutaneous fat, and graying of the hair appearing before one year of age. Weight loss, anemia, osteoporosis, thymic involution, testicular atrophy and reduced fertility have also been observed in these mice [3], [4]. Accordingly, Polg mutator mice also have shortened lifespans with medians of 41 [4] and 59 [3] weeks compared to greater than 2 years reported for wild type mice [3].

Several studies have attempted to understand the relationship between mtDNA alterations and the progeroid phenotype of Polg mice. Trifunovic et al identified the presence of large (∼12 kb) linear mtDNA molecules resulting from deletions in mtDNA and an increased mtDNA mutation load in tissues from Polg mutator mice [4]. Higher levels of mtDNA mutations were also originally reported by Prolla and colleagues [3]. However, the question of whether mtDNA mutations or deletions are the driving force of the progeroid phenotype is unsettled [5]–[10].

The thirteen proteins encoded by mtDNA are all members of mitochondrial respiratory chain complexes and it might therefore be predicted that a major outcome of mtDNA mutations/deletions would be respiratory chain dysfunction. Supporting this concept, embryonic fibroblasts isolated from Polg mutator mice have lower mitochondrial respiration compared to controls [11]. Several studies have also demonstrated the presence of cytochrome c oxidase (COX) negative cells in the brains of Polg mutator mice, indicating respiratory chain dysfunction [5], [12], [13]. The steady state levels of two proteins that make up the COX complex (mtDNA encoded Mtco2 and nuclear DNA (nDNA) encoded COX4i1) have been reported to be decreased in liver and heart mitochondria isolated from mutator mice [8]. In addition, levels of fully assembled complexes I, III, and IV were observed to be decreased in mutator liver mitochondria along with complex I activity [8]. Decreased abundance of the nuclear encoded 39 kDa subunit of complex I (NDUFA9) and the mtDNA encoded Mtco1 has been observed in brain mitochondrial extracts from mutator mice [12]. Other nDNA and mtDNA encoded respiratory chain subunits are also decreased in several tissues of the Polg mutator mice including brain [14]. One proposed mechanism for the decrease of both mtDNA and nDNA encoded subunits of respiratory chain complexes in mutator mouse liver mitochondria is that point mutations in mtDNA encoded subunits lead to misassembled complexes that are subject to rapid protein turnover [8]. There are also reports of decreases in the mRNA coding for the master regulator of mitochondrial biogenesis PGC-1α [14] and several nDNA encoded subunits of complexes I, III, and IV [15] in muscle from the mutator mice, although decreased PGC-1α expression in brain has not been reported [14].

These previous results therefore suggest that there are, as might be expected, consistent changes in proteins encoded by the mtDNA in Polg mutator mice but have not clearly addressed whether, first, there are more widespread protein changes including nuclear encoded mitochondrial proteins, and, second, these are accompanied by mRNA changes. To address these questions, we analyzed the brain mitochondrial proteome using quantitative proteomics and the brain transcriptome using RNA Sequencing (RNA-Seq) in aged Polg mutator mice and their littermates. Surprisingly, we found a strong effect on nuclear encoded mitochondrial proteins without obvious changes in gene expression.

## Materials and Methods

### Polg Mutator Mice

This study was carried out in strict accordance with the recommendations in the Guide for the Care and Use of Laboratory Animals of the National Institutes of Health. The protocol was approved by the Institutional Animal Care and Use Committees of the US National Institute of Child Health and Human Development (Animal study protocol number 12-059).

Polg mutator mice (Polg^D257A^ knock-in) were originally generated by Dr. Thomas Prolla at the University of Wisconsin [3]. Polg^WT/WT^, Polg^WT/D257A^, and Polg^D257A/D257A^ were generated by breeding Polg^WT/D257A^ heterozygotes. Animals had access to food and water *ad libitum*. At one year of age, mice were sacrificed and their brains were dissected on ice. The cerebellum was removed and the cerebrum was bisected along the longitudinal fissure. The cerebellum and one cerebral hemisphere were frozen on dry ice, while mitochondria were immediately prepared from the other cerebral hemisphere.

### Preparation of Brain Mitochondria

Cerebral tissue was homogenized in isolation buffer (0.225 M Mannitol, 0.05 M Sucrose, 0.005 M HEPES pH 7.3) supplemented with protease inhibitors (Roche). The homogenate was centrifuged at 2000x*g* for 3 min at 4°C and the pellet was resuspended in isolation buffer and the centrifugation step was repeated. The combined S2000 was centrifuged (12000x*g*, 8 min, 4°C) and the mitochondrial pellet was resuspended in isolation buffer supplemented with 0.02% digitonin to rupture synaptosomes prior to centrifugation (12000x*g*, 10 min, 4°C). The resulting mitochondrial pellet was washed in isolation buffer without digitonin and centrifuged at 12000x*g* for 10 min at 4°C. Mitochondrial pellets were then immediately frozen and stored at −80°C until use.

### Preparation of Mitochondrial Protein Extracts

Frozen mitochondrial pellets were thawed on ice and resuspended in iTRAQ lysis buffer (0.3 M HEPES pH 8.0, 2% w/v CHAPS, 1 mM EDTA) by vortexing. Samples were vortexed periodically for 15 min and kept on ice prior to centrifugation (16,000x*g*, 10 min, 4°C). The supernatants were used in iTRAQ and Western blotting experiments. Protein content was determined using the 660 nm protein assay reagent (Pierce #22660) using bovine serum albumin for standardization.

### Preparation of Cerebellar Protein Extracts

Frozen cerebella were homogenized in isolation buffer and then mixed with an equal volume of 2x iTRAQ lysis buffer in order to treat the samples in a similar manner as the cerebral mitochondria were treated. The homogenates were further processed in the same manner as described for the mitochondrial extracts.

### iTRAQ Labelling

4 control samples and 7 mutant samples were analyzed in two separate iTRAQ experiments. 40 μg of each control sample was taken and pooled together to make a combined reference sample. This reference sample was labeled with iTRAQ reagent 113 and included in both LC-MS/MS runs, which allows for accurate comparisons across multiple LC-MS/MS runs. iTRAQ labeling was carried out using iTRAQ Reagent 8-Plex kit (AB Sciex, Foster City, CA) and followed the manufacturer's protocol with minor modifications. For each sample, 40 μg protein was used, and the concentration was adjusted to 1 μg/μl with iTRAQ dissolution buffer, reduced with 2 μl Reducing Reagent (TCEP solution) for 60 min at 60°C, alkylated with 1 μl Cysteine Blocking Reagent (MMTS solution) at RT for 20 min, and digested overnight with sequence grade modified trypsin (Promega, Madison, WI) at 37°C with a trypsin to protein ratio of 1∶50 (w/w). Each iTRAQ 8-plex reagent was dissolved in 150 μl isopropanol, vortexed, and added to each sample. The sample/reagent mixture was incubated at room temperature for 3 hr. Labeled peptides were combined, and then desalted using an Oasis HLB 200 mg cartridge (Waters, Milford, MA).

### 2D LC-MS/MS Analysis

iTRAQ labeled peptides were fractionated on a 4.6 mm ID TSKGel Amide 80 HILIC column (Tosoh) using the Agilent 1100 Series LC (Agilent, Santa Clara, CA). Samples were suspended in mobile phase B (95% acetonitrile and 0.1% formic acid) and peptides were eluted at 700 μl/min by increasing the mobile phase A (95% water and 0.1% FA) amount from 3% to 50% over 30 min. A total of 24 fractions were collected. MALDI-MS was used to analyze the peptide complexity of each fraction. Fractions containing only minimal numbers of peptides were pooled together, resulting in a total of 14 fractions selected for further analysis. Each fraction was then analyzed by a nano-LC-MS/MS system with an Ultimate 3000 HPLC (Thermo-Dionex) connected to an Orbitrap Elite mass spectrometer (Thermo Fisher Scientific) via an Easy-Spray ion source (Thermo Fisher Scientific). An ES800 Easy-Spray column (75 μm inner diameter, 15 cm length, 3 μm C18 beads) was used. Peptides were separated with a 100 min gradient of 2-X% mobile phase B (mobile phase A: 98% water, 0.1% formic acid; mobile phase B: 98% acetonitrile, 0.1% formic acid, X varies from 20 to 40 for different HILIC fractions).The Thermo Scientific Orbitrap Elite mass spectrometer was operated in positive nano-electrospray mode. Both MS and MS/MS data were acquired in the orbitrap with profile mode. The resolution of the survey scan (m/z 300–1600) was set at 60 k at m/z 400 with a target value of 1×10^6^ ions. The four most intense ions from the preview survey scan were sequenced by high energy collision dissociation fragmentation (HCD). HCD was performed using collision energy of 38%. The resolution of the MS/MS scan was set at 15 k.

### Peptide Identification and Quantitation

Xcalibur RAW files were converted to peak list files in mgf format using Mascot Distiller (version 2.4.3.3). The parameters for data processing were: always uncentroid, no smooth, applied baseline correction, no deisotoping for m/z 112.9–121.4 range. The threshold for peak picking was S/N 3 for MS data and S/N 2 for MS/MS data. A database search was submitted through Mascot Daemon (version 2.4.0) to Mascot Server (version 2.4) against the Sprot Mouse database (16,662 sequences). In all database searches, trypsin was specified as the enzyme, and up to 2 missed cleavages were allowed. Precursor and fragment mass tolerance were set at 20 ppm and 0.2 Da, respectively. iTRAQ8plex (N-term) and iTRAQ8plex (K) were searched as fixed modifications, Methylthio (C), Oxidation (M), and iTRAQ8plex (Y) were set as variable modifications. A decoy search was also performed. False detective rate (FDR) was set at <1% when reporting the protein identification results. The “iTRAQ 8plex” method was selected when submitting the search, so quantitation of iTRAQ-labeled peptides was also performed by Mascot software used for the protein identification. All MS/MS files obtained from 14 LC-MS/MS runs were merged into a single search. Peptide ratios for each biological sample were obtained by dividing the intensity of that sample's iTRAQ label by the intensity of the label of the pooled reference in channel 113. These peptide ratios were normalized so that the median ratio for each sample was 1, and outliers were removed using MASCOT's automatic method. Protein ratios were calculated as the weighted average of peptide ratios and were reported only when 2 or more peptides matched to that protein with a score higher than the homology threshold. Only unique peptides were used for protein ratio calculations. Only proteins that had ratios >0 for each sample's iTRAQ channel and were present in both LC-MS/MS datasets were selected for statistical analysis. Ratios from the 1060 proteins present in both datasets were log_2_ transformed then control and mutator groups were compared using Welch's t test. P values were adjusted using the Benjamini-Hochberg method in order to correct for multiple testing.

Protein ratios from both LC-MS/MS runs and statistical analysis for all 1060 proteins identified in both runs can be found in Supplemental File 1.

### Western Blotting

Protein extracts from the 11 male mice used for iTRAQ analysis were separated using SDS-PAGE and transferred to PVDF membranes. Membranes were blocked for 1 hr using Odyssey Blocking Buffer (LI-COR) and probed overnight with primary antibody diluted in a 1∶1 mixture of Odyssey Blocking Buffer and TBS+0.1% Tween-20. The primary antibodies used were against HSP60 (Abcam 46798, 1∶2000), DJ-1 (Abcam 169520, 1∶2000-1∶4000), COX4i1 (Abcam 16056, 1∶2000-1∶100,000), NDUS8 (Abcam 110245, 1∶2000), Mtco2 (Abcam 110258, 1∶1000), and Actin (Sigma A1978, 1∶400,000). Secondary antibodies (LI-COR 926-32210 and 926–68023,) were diluted 1∶15000 in blocking buffer and incubated with membranes for 1 hr at RT. Blots were imaged using a LI-COR Odyssey CLx Infrared Imaging System and quantification was performed using LI-COR Image Studio software.

### RNA extraction and sequencing

Total RNA was extracted from frozen cerebral tissue with TRIzol (Invitrogen) using a tissue homogenizer. RNA quality was determined using the Agilent 2100 Bioanalyzer RNA Nano Chip (Mean RIN = 9.22, RIN Range = 7.8–9.6). We purified poly(A)+RNA from 4 μg total RNA using poly-T oligo attached magnetic beads and synthesized cDNA libraries using the TruSeq RNA sample preparations kit with random priming (Illumina cat. no. RS-122-2001) as per the manufacturer's protocol. The 11 libraries were multiplexed (6 samples per pool) and 3.5 pmol of pooled cDNA was run on a single flowcell lane. 100-bp paired end sequences were generated using an Illumina Hi-Seq sequencer.

### Statistical analysis of RNA-Seq data

This study utilized the high-performance computational capabilities of the Biowulf Linux cluster at the National Institutes of Health, Bethesda, Md. (http://biowulf.nih.gov). RNA-Seq data was handled according to our previously published methods [16]. We used the Illumina pipeline to analyze the images and extract base calls for fastq file generation. Overall quality and total read counts can be found in Supplementary File 2. Fastq files were aligned to the mm9 mouse reference genome using Tophat (v2.0.6) and Bowtie (2.02.0) using the ensembl gtf (Mus_musculus.NCBIM37.61) to build bowtie indexes. Reads were annotated and quantified to a given gene using the Python module HT-SEQ using the same ensembl gtf to provide reference boundaries.

For gene expression analysis, we used the R/Bioconductor package DESeq [17]. The count data was normalized for library size and then transformed using variance-stabilization. Poisson distributions of normalized counts for each transcript were compared across mutator and control groups using a negative binomial test and p values were adjusted using the Benjamini-Hochberg procedure.

For alternative exon utilization analysis, the ensembl gtf was flattened using the python script written by Simon Anders to create the appropriate counting bins needed for downstream analysis. We used the R/Bioconductor package DEXSeq [18] to adjust for library size difference, to estimate variance, and then the Poisson distributions of normalized counts for each exon were compared across mutator and control groups using a negative binomial test. Multiple testing was corrected for using the Benjamini-Hochberg procedure.

The RNA-Seq data generated in this experiment has been submitted to GEO and given accession number GSE55188. Output from DESeq and DEXSeq can be found in Supplemental File 2.

## Results

### Protein Analysis

**Figure 1:**
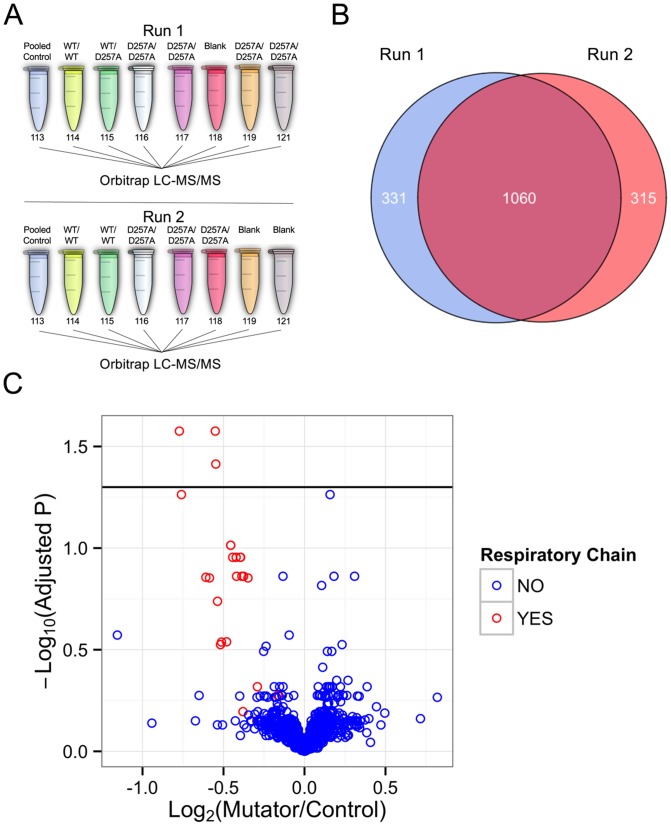
Proteomic analysis of the Polg mutator mouse brain. A) Schematic of iTRAQ labeling and LC-MS/MS experiment. A pool of the four control (3 WT/WT and 1 WT/D257A) brains was used as a reference in both LC-MS/MS runs and allowed for cross-run comparison. B) Venn diagram displaying the total number of proteins identified and quantified in each LC-MS/MS run. C) Volcano plot of the 1060 proteins quantified in both LC-MS/MS runs (n = 4 Control, n = 7 Mutator, Welch T-test p values adjusted using Benjamini-Hochberg method to control FDR). Data points are colored by membership in the Uniprot “Respiratory Chain” gene ontology group. The horizontal line shows the preset p value cutoff for statistical significance (FDR adjusted p<0.05).

We performed a quantitative proteomics experiment using mitochondrial enriched protein extracts from the brains of one-year-old Polg mutator mice (D257A/D257A) and their control (WT/WT and WT/D257A) littermates. At the time of sacrifice, all Polg mutator mice displayed a progeroid phenotype and weighed less than control littermates (Fig. S1). Protein samples were labeled with isobaric tags for relative and absolute quantitation (iTRAQ) 8-plex reagents and analyzed using two separate liquid chromatography coupled with tandem mass spectrometry (LC-MS/MS) runs against a pooled reference sample in each run (Fig. 1A). Including the same sample as the reference in both LC-MS/MS runs allows for comparisons to be made across runs as others have successfully done before [19]. Approximately 1400 proteins were identified and quantified in each LC-MS/MS experiment, with 1060 in common between the two (Figure 1B). A gene ontology analysis using DAVID demonstrated that these 1060 proteins were enriched with proteins associated with the cellular component term mitochondrion (n = 289 proteins with GOTERM_CC mitochondrion, Benjamini-Hochberg adjusted p value  = 1.8×10^−75^) [20]. Statistical analysis of these 1060 proteins revealed that three proteins (NDUS4, Mtco2, and NDUC2) were significantly (*P*<0.05 after FDR correction at 5%) decreased in the brains of the Polg mutator mice compared to controls (Fig. 1C). Interestingly, these three proteins are all subunits of respiratory chain complexes; NDUS4 and NDUC2 are nuclear, Mtco2 is encoded for by mtDNA. When we subset the proteomics data by membership in the Uniprot group “Respiratory Chain” we noticed that while many proteins did not reach the preset requirement for statistical significance of FDR adjusted p<0.05, there was a clear trend for decrease across this group (Fig. 1C). The top 20 differentially expressed proteins based on p-value are clearly enriched with respiratory chain proteins (16/20 proteins; Table 1). Fifteen of the 45 proteins that comprise Complex I are represented on this list. Although not significant under our preset criteria for statistical significance, it should be noted that all 20 proteins listed in Table 1 would be statistically significant using a relaxed FDR of 15%. Taken together, these data suggest that the primary change in the brain mitochondrial proteome of Polg mutator mice is a decrease of respiratory chain proteins.

**Table 1:** List of the top twenty differentially expressed proteins from aged Polg mutator mouse brain based on p value from iTRAQ analysis.

Accession	Peptides^1^		Sequence Coverage (%)		Log_2_ Fold Change^2^	Unadjusted P^3^	Adjusted P^4^	Respiratory Chain
	Run 1	Run 2	Run 1	Run 2				
NDUS4_MOUSE	3 (21)	4 (12)	14.3	28.6	−0.551	0.00004	0.027	YES
COX2_MOUSE	3 (26)	2 (22)	10.6	7.5	−0.772	0.00005	0.027	YES
NDUC2_MOUSE	1 (9)	1 (7)	5.0	5.8	−0.548	0.00011	0.039	YES
AP2A1_MOUSE	7 (19)	9 (33)	13.2	15.5	0.158	0.00022	0.055	NO
NDUA4_MOUSE	2 (21)	2 (17)	20.7	20.7	−0.760	0.00026	0.055	YES
NDUS7_MOUSE	1 (6)	2 (10)	4.0	8.5	−0.455	0.00060	0.097	YES
NDUA9_MOUSE	3 (16)	4 (19)	9.5	15.1	−0.456	0.00064	0.097	YES
NDUB7_MOUSE	3 (12)	6 (34)	30.6	42.3	−0.443	0.00084	0.111	YES
NDUS8_MOUSE	2 (13)	2 (8)	7.5	7.5	−0.422	0.00107	0.111	YES
NDUBA_MOUSE	2 (8)	2 (9)	9.6	14.8	−0.397	0.00107	0.111	YES
NDUV2_MOUSE	4 (18)	5 (17)	20.6	20.2	−0.394	0.00115	0.111	YES
CRYM_MOUSE	1 (4)	1 (5)	2.6	2.9	0.182	0.00162	0.138	NO
NDUS3_MOUSE	3 (13)	4 (21)	12.2	19.4	−0.387	0.00181	0.138	YES
GSTM1_MOUSE	4 (31)	4 (31)	29.4	23.9	0.309	0.00191	0.138	NO
KCRB_MOUSE	7 (82)	6 (94)	28.3	26.8	−0.133	0.00209	0.138	NO
NDUAD_MOUSE	3 (16)	1 (5)	24.3	7.6	−0.419	0.00220	0.138	YES
NDUS1_MOUSE	6 (30)	6 (32)	13.5	12.4	−0.376	0.00221	0.138	YES
NDUB3_MOUSE	1 (2)	1 (2)	6.0	6.7	−0.609	0.00236	0.139	YES
NDUS5_MOUSE	1 (3)	1 (3)	11.3	11.3	−0.587	0.00260	0.140	YES
NDUBB_MOUSE	1 (3)	3 (6)	13.2	27.1	−0.349	0.00265	0.140	YES

1 The total number of unique peptide sequences detected are shown outside of parentheses. The total number of replicated unique peptides used for quantification is shown within parentheses.

2 Log_2_ (Mutator/Control).

3 Welch T-Test P-value.

4 Welch T-Test P-value adjusted using Benjamini-Hochberg method to control FDR.

**Figure 2:**
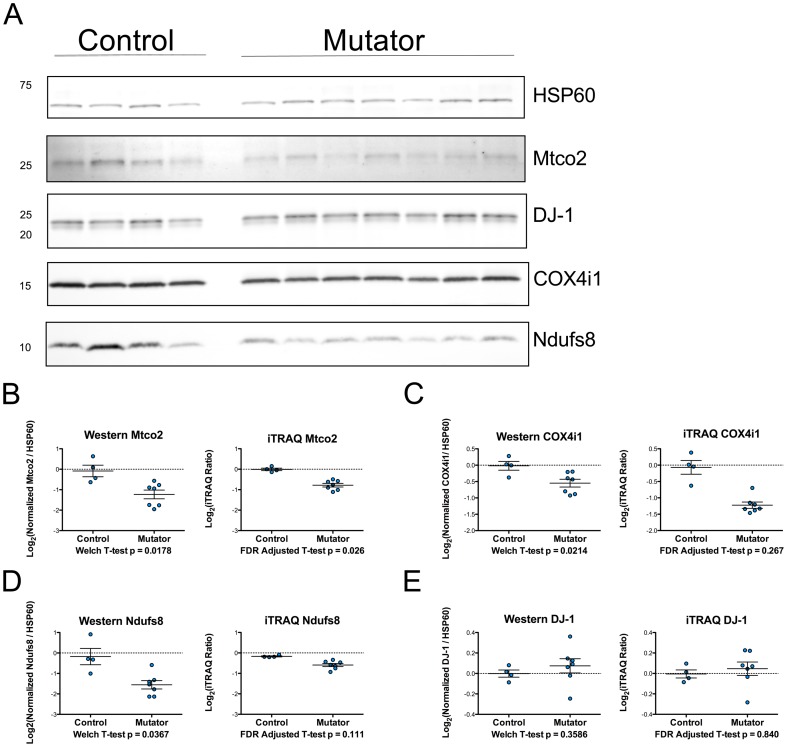
Validation of iTRAQ data using Western blots. A) Western blots for the loading control HSP60 and the proteins of interest Mtco2, DJ-1, COX4i1, and Ndufs8. Proteins samples analyzed were from the 11 male mice used in the iTRAQ analysis. B–E) Quantitative data from Western blots and iTraq experiment for proteins of interest. Western blot data was normalized to the mean of the controls and log_2_ transformed prior to comparison using Welch T-tests. HSP60 was used as a loading control to normalize the data.

To validate the findings from the iTRAQ experiment, we performed Western blots for several proteins of interest in the mitochondrial extracts (Figure 2). We measured four proteins with a range of p values from the iTRAQ data that had commercially available antibodies. Ndufs8 (iTRAQ unadjusted p = 0.00107, iTRAQ FDR adjusted p = 0.111), Mtco2 (iTRAQ unadjusted p = 0.00005, iTRAQ FDR adjusted p = 0.027), and COX4i1 (iTRAQ unadjusted p = 0.006, iTRAQ FDR adjusted p = 0.261) were confirmed to be significantly different between the two groups (Ndufs8 p = 0.0367, Mtco2 p = 0.0178, and COX4i1 p = 0.0214). DJ-1, which was not different between the two groups in the iTRAQ dataset (iTRAQ unadjusted p = 0.51, iTRAQ FDR adjusted p = 0.83) was confirmed to be unchanged via Western blot.

**Figure 3:**
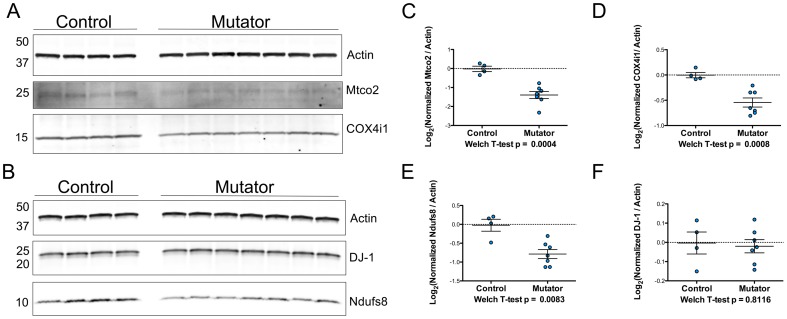
Western blot analysis of cerebellar proteins of interest. A–B) Actin, Mtco2, COX4i1, Ndufs8, and DJ-1 protein levels were measured in cerebellar protein extracts from the mice used in the iTRAQ experiment. C–F) Quantitation of Western blots (n = 4 control, n = 7 mutator). Data was normalized to the mean of the controls and log_2_ transformed. Welch T-tests were used to compare groups and the p-values are listed beneath the graphs. Actin was used as a loading control to normalize data.

We also used Western blots to determine if some of the findings from the iTRAQ experiment could be observed in other neural tissue from the same mice. We found that the levels of Mtco2, COX4i1, and Ndufs8 were all significantly decreased in the cerebella of the mutator mice (Figure 3A–E). DJ-1 was found to not be significantly different between groups (Figure 3B and 3F). Collectively, these results validate our iTRAQ data and suggest that at least a subset of the differences that did not pass our preset cutoff for significance (FDR adjusted p<0.05) are genuine.

### RNA-Seq

**Figure 4:**
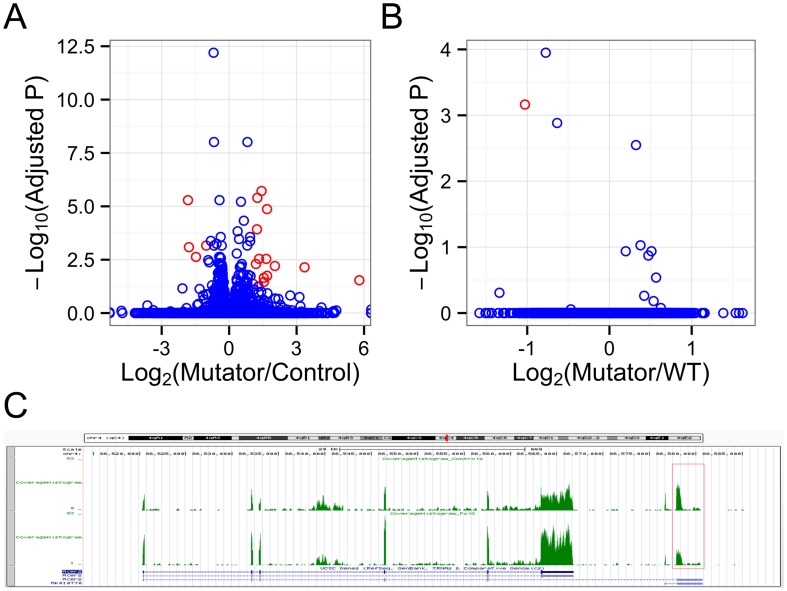
RNA-Seq data. A) Volcano plot of DESEQ results showing gene expression changes. Genes that were changed greater than 2-fold and statistically significant are colored red (n = 4 controls (3 WT/WT and 1 WT/D257A), n = 7 mutator). B) Volcano plot of DEXSEQ results showing differential exon usage. Exons that were statistically significant and changed greater than 2-fold are shown in red (n = 3 WT/WT, n = 7 mutator). C) UCSC Genome Browser read count histogram for Acer2. Overall expression of this gene was significantly increased in Polg mutator mice, but usage of an alternative 5′UTR (boxed in light red) was significantly decreased in the mutator mice.

We performed RNA-Seq using mRNA extracted from the same cohort of 11 male aged mice used in the iTRAQ experiment. From 28,100 genes identified using RNA-Seq, only 18 were expressed differently between Polg mutator mice and controls (FDR adjusted p<0.05 and Fold Change >2)(Fig. 4A and Table 2). At the exon level, only exon 9 (an alternate 5′UTR) of the gene *Acer2* was significantly decreased in the brains of the Polg mutator animals (FDR adjusted p<0.05 and Fold Change >2)(Fig. 4B–C). Of note, total Acer2 mRNA was increased in the mutator mice (Fig. 4A and Table 2). Taken together, our data suggests that despite having extensive coverage there are relatively few differences in mRNA expression and differential exon usage in the brains of aged Polg mutator mice.

**Table 2:** Differentially expressed mRNA transcripts in the brains of aged Polg mutator mice as determined by RNA-seq.

Gene ID	Base Mean	Base Mean Control	Base Mean Mutator	Log_2_ Fold Change^1^	P Value	Adjusted P Value^2^
Acer2	1031.46	493.30	1338.98	1.44	3.40E-10	1.91E-06
Smad6	226.89	120.55	287.66	1.25	8.54E-10	4.00E-06
Igk-C	23.72	43.74	12.29	−1.83	1.46E-09	5.14E-06
Edn1	268.51	111.40	358.29	1.69	4.79E-09	1.35E-05
Vwf	2585.26	1386.79	3270.11	1.24	5.10E-08	0.00011941
Klk6	279.04	411.95	203.08	−1.02	4.93E-07	6.88132E-04
Gm16233	17.26	31.51	9.11	−1.79	6.99E-07	8.18628E-04
Gm5535	25.41	42.89	15.42	−1.48	2.33E-06	0.002352071
Bst1	28.33	12.01	37.66	1.65	3.19E-06	0.002891414
Treml2	34.48	17.56	44.14	1.33	3.13E-06	0.002891414
Cxcl12	6368.84	3505.96	8004.77	1.19	6.48E-06	0.005040626
A730020M07Rik	17.49	5.89	24.12	2.03	9.10E-06	0.006235073
Ppbp	23.88	3.48	35.53	3.35	1.18E-05	0.007205275
Gm13340	512.82	212.30	684.55	1.69	3.90E-05	0.017985475
Angpt2	248.51	112.13	326.45	1.54	5.42E-05	0.022402713
Tubb1	12.19	0.34	18.96	5.79	7.43E-05	0.028998941
Rtp1	99.26	44.67	130.46	1.55	9.68E-05	0.034882988
5430416O09Rik	58.56	34.70	72.19	1.06	1.28209E-04	0.04238892

1 Log_2_ (Mutator/Control).

2 P-value adjusted using Benjamini-Hochberg method to control the FDR.

### Comparison of Protein and mRNA

**Figure 5:**
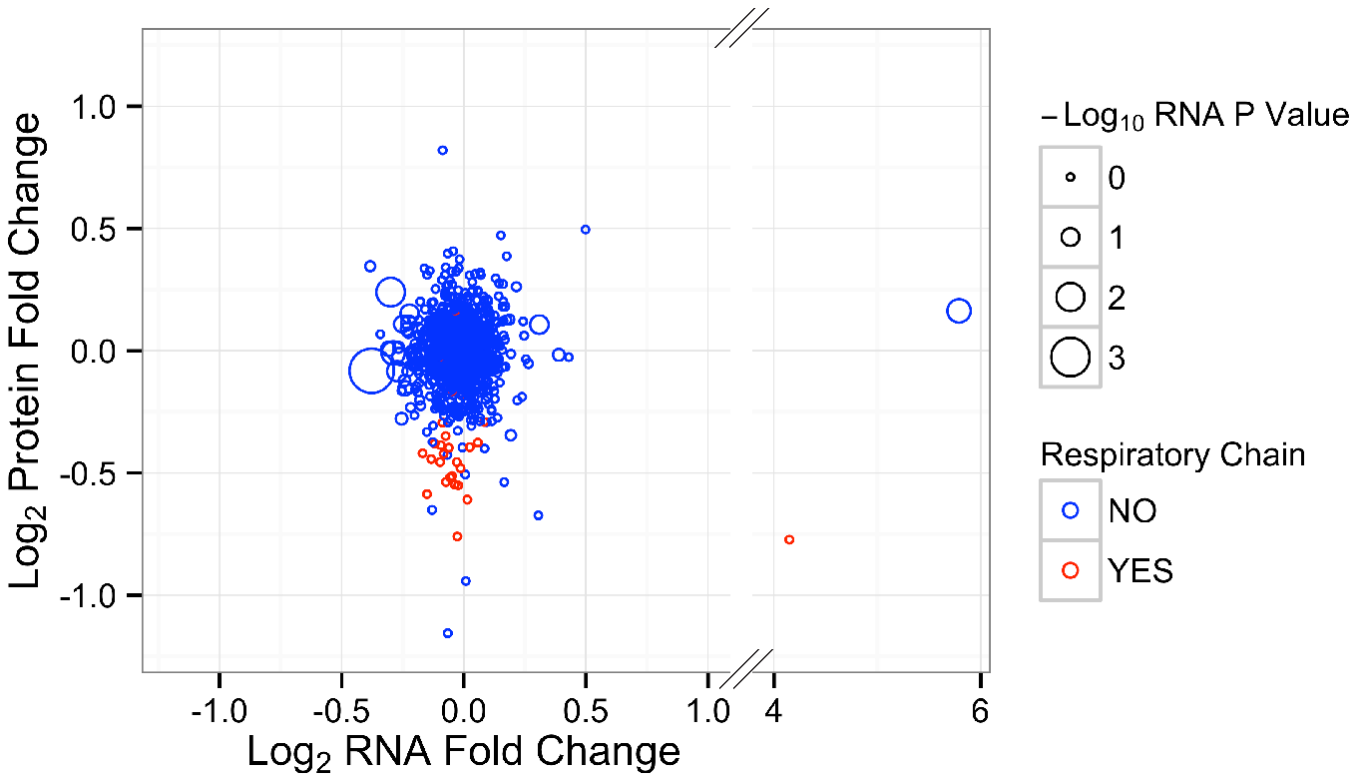
Comparison of iTRAQ and RNA-Seq datasets. The fold changes of 1001 proteins and gene transcripts that were measured using both iTRAQ and RNA-Seq were plotted against each other. Data points are colored by membership in the Uniprot gene ontology group “Respiratory Chain” and they are sized according to the adjusted p-value from the RNA-Seq dataset. The x-axis was broken to allow for easier visualization of all the data. The two data points to the right of the break represent Mtco2 and Tubb1 and have adjusted p values from the RNA-Seq experiment of 1 and 0.0289, respectively.

In order to understand likely mechanisms by which protein level differences might occur, we next compared the mRNA and protein data. We plotted the fold change of protein versus the fold change of mRNA for 1001 proteins present in both the iTRAQ and RNA-Seq datasets (Figure 5). All of the respiratory chain proteins that were measured in the iTRAQ experiment had their corresponding mRNA levels measured in the RNA-Seq experiment. However, none of the mRNAs for these proteins were differentially expressed. Furthermore, we observed no correlation between protein fold change and mRNA fold change (linear regression model r^2^ = 0.001; p = 0.7585). These results suggest that mRNA changes do not explain protein level differences and, by inference, that the altered mitochondrial protein levels occur post-translationally.

## Discussion

Here, we have reported the results of the first systematic analysis of the mitochondrial-enriched brain proteome of the Polg mutator mouse. This approach identified three mitochondrial respiratory chain proteins that were significantly decreased in the brains of Polg mutator mice, and suggested that many other respiratory chain proteins are also decreased in their brains. Combining proteomic analyses with RNA-Seq revealed that there are few changes in mRNA expression and differential exon usage in the brains of the Polg mutator mouse. In contrast to the changes in abundance of respiratory chain proteins we measured in our proteomics experiment, we observed no changes in the expression levels of mRNAs for both mtDNA and nuclear encoded respiratory chain proteins. Collectively, these results show that there are specific changes in the mitochondrial proteome at the post-translational level of regulation.

Our proteomics data support several previous observations from studies of this progeroid model. Decreased COX activity based staining has been observed in the brains, hearts, and duodenums of Polg mutator mice [4], [5], [13]. Muscle from the mutator mice has also been shown to have decreased abundance of complexes I, III, and IV [15] and decreased COX assembly [14] using Blue-Native PAGE. Western blotting using antibodies for specific respiratory chain subunits has also shown decreased levels of those proteins in multiple tissues including brain [8], [14]. However, the current dataset is the first to demonstrate these changes using a non-targeted mass spectrometry technique. Using this approach, we have demonstrated that the loss of complex I subunits in the mutator mice encompasses many more proteins than has been previously demonstrated. Importantly, these changes include not only mtDNA- but also nuclear-encoded proteins. We hypothesize that the primary defect in mtDNA can then lead to effects on mitochondrial complexes more widely.

Our data does not support one possible explanation for these differences, namely that mRNA expression is altered. Previous studies of the transcriptome in Polg mutator mice have indicated a variety of results. One study using microarrays found significant changes in the expression of 97 mRNA transcripts in the muscle of aged Polg mutator mice [15]. The authors reported the downregulation of numerous nuclear encoded transcripts that code for electron transport chain proteins [15]. Others have found significant downregulation of the transcripts for PGC-1α, mitochondria transcription factor A (Tfam), COX4i1, and several other mtDNA and nuclear encoded genes using qPCR in muscle from Polg mutator mice although the mRNA for PGC-1α was not changed in brain [14]. In contrast to muscle, we found minimal changes in the brains of the mice at the mRNA transcript level. There are therefore likely tissue specific differences in mRNA expression levels but, importantly, mRNA changes do not explain protein level changes in the brain from the same animals.

One limitation of our dataset, and of large scale approaches more generally, is that the need for multiple test correction can lead to type II statistical errors. Our finding that the differences observed in COX4i1 and Ndufs8 levels between the two groups using the iTRAQ analysis could also be seen by Western blotting in the cerebral mitochondrial samples used for proteomic analysis and also in protein extracts from the cerebella of the same mice suggests that even within sub-significant hits there may be true differences in protein levels. Whether other modest changes in protein levels that did not reach significance in the iTRAQ experiments are also genuinely different between control and Polg mutator mice remains to be established.

Taken together, our data suggest that the decreases in abundance of mitochondrial respiratory chain proteins is not explained by alterations in the levels of their corresponding mRNA transcripts in the brain. We therefore infer that the mechanism of loss of mitochondrial complex subunits is at the protein level. A likely explanation for this is that mitochondrial complexes are destabilized by mutations in the mtDNA encoded proteins which then fail to assemble properly with their nuclear encoded counterparts. This prediction, that arises from combined analysis of two different large scale approaches, can be tested in future studies.

## Supporting Information

Figure S1 **Polg mutator mice used in this study.** A) Two male one year old littermates used in this study (WT/WT in background and Polg mutator in foreground). All of the mutator mice developed the characteristic progeroid phenotype. B) Mutator mice were significantly smaller than the controls used in the study at one year of age (control n = 4 (3 WT/WT and 1 WT/D257A) and mutator n = 7, * indicates t test p<0.05).
(TIF)

File S1 **Complete iTRAQ Dataset.**
(XLSX)

File S2 **RNA-Seq Analysis Data.**
(XLSX)

## References

[1] Bratic A, Larsson N-G (2013) The role of mitochondria in aging. J Clin Invest 123: 951–957 10.1172/JCI64125 23454757 PMC3582127

[2] Taylor RW, Turnbull DM (2005) MITOCHONDRIAL DNA MUTATIONS IN HUMAN DISEASE. Nat Rev Genet 6: 389–402 10.1038/nrg1606 15861210 PMC1762815

[3] Kujoth GC, Hiona A, Pugh TD, Someya S, Panzer K, et al (2005) Mitochondrial DNA mutations, oxidative stress, and apoptosis in mammalian aging. Science 309: 481–484 10.1126/science.1112125 16020738

[4] Trifunovic A, Wredenberg A, Falkenberg M, Spelbrink JN, Rovio AT, et al (2004) Premature ageing in mice expressing defective mitochondrial DNA polymerase. Nature 429: 417–423 10.1038/nature02517 15164064

[5] Vermulst M, Wanagat J, Kujoth GC, Bielas JH, Rabinovitch PS, et al (2008) DNA deletions and clonal mutations drive premature aging in mitochondrial mutator mice. Nat Genet 40: 392–394 10.1038/ng.95 18311139

[6] Vermulst M, Bielas JH, Kujoth GC, Ladiges WC, Rabinovitch PS, et al (2007) Mitochondrial point mutations do not limit the natural lifespan of mice. Nat Genet 39: 540–543 10.1038/ng1988 17334366

[7] Williams SL, Huang J, Edwards YJ, Ulloa RH, Dillon LM, et al (2010) The mtDNA mutation spectrum of the progeroid Polg mutator mouse includes abundant control region multimers. Cell Metab 12: 675–682 10.1016/j.cmet.2010.11.012 21109200 PMC3175596

[8] Edgar D, Shabalina I, Camara Y, Wredenberg A, Calvaruso MA, et al (2009) Random point mutations with major effects on protein-coding genes are the driving force behind premature aging in mtDNA mutator mice. Cell Metab 10: 131–138 10.1016/j.cmet.2009.06.010 19656491

[9] Kraytsberg Y, Simon DK, Turnbull DM, Khrapko K (2009) Do mtDNA deletions drive premature aging in mtDNA mutator mice? Aging Cell 8: 502–506 10.1111/j.1474-9726.2009.00484.x 19416127 PMC3137638

[10] Tyynismaa H, Mjosund KP, Wanrooij S, Lappalainen I, Ylikallio E, et al (2005) Mutant mitochondrial helicase Twinkle causes multiple mtDNA deletions and a late-onset mitochondrial disease in mice. Proc Natl Acad Sci U S A 102: 17687–17692 10.1073/pnas.0505551102 16301523 PMC1308896

[11] Trifunovic A, Hansson A, Wredenberg A, Rovio AT, Dufour E, et al (2005) Somatic mtDNA mutations cause aging phenotypes without affecting reactive oxygen species production. Proc Natl Acad Sci U S A 102: 17993–17998 10.1073/pnas.0508886102 16332961 PMC1312403

[12] Ahlqvist KJ, Hämäläinen RH, Yatsuga S, Uutela M, Terzioglu M, et al (2012) Somatic progenitor cell vulnerability to mitochondrial DNA mutagenesis underlies progeroid phenotypes in Polg mutator mice. Cell Metab 15: 100–109 10.1016/j.cmet.2011.11.012 22225879

[13] Ross JM, Öberg J, Brené S, Coppotelli G, Terzioglu M, et al (2010) High brain lactate is a hallmark of aging and caused by a shift in the lactate dehydrogenase A/B ratio. Proc Natl Acad Sci 107: 20087–20092 10.1073/pnas.1008189107 21041631 PMC2993405

[14] Safdar A, Bourgeois JM, Ogborn DI, Little JP, Hettinga BP, et al (2011) Endurance exercise rescues progeroid aging and induces systemic mitochondrial rejuvenation in mtDNA mutator mice. Proc Natl Acad Sci U S A 108: 4135–4140 10.1073/pnas.1019581108 21368114 PMC3053975

[15] Hiona A, Sanz A, Kujoth GC, Pamplona R, Seo AY, et al (2010) Mitochondrial DNA mutations induce mitochondrial dysfunction, apoptosis and sarcopenia in skeletal muscle of mitochondrial DNA mutator mice. PloS One 5: e11468 10.1371/journal.pone.0011468 20628647 PMC2898813

[16] Dillman AA, Hauser DN, Gibbs JR, Nalls MA, McCoy MK, et al (2013) mRNA expression, splicing and editing in the embryonic and adult mouse cerebral cortex. Nat Neurosci 16: 499–506 10.1038/nn.3332 23416452 PMC3609882

[17] Anders S, Huber W (2010) Differential expression analysis for sequence count data. Genome Biol 11: R106 10.1186/gb-2010-11-10-r106 20979621 PMC3218662

[18] Anders S, Reyes A, Huber W (2012) Detecting differential usage of exons from RNA-seq data. Genome Res 22: 2008–2017 10.1101/gr.133744.111 22722343 PMC3460195

[19] Wu L, Candille SI, Choi Y, Xie D, Jiang L, et al (2013) Variation and genetic control of protein abundance in humans. Nature 499: 79–82. 23676674 10.1038/nature12223 PMC3789121

[20] Huang DW, Sherman BT, Lempicki RA (2008) Systematic and integrative analysis of large gene lists using DAVID bioinformatics resources. Nat Protoc 4: 44–57 10.1038/nprot.2008.211 19131956

