# Supplementary figures and images for: Post-Translational Decrease in Respiratory Chain Proteins in the Polg Mutator Mouse Brain
Source: PLoS One. 2014 Apr 10;9(4):e94646. doi: 10.1371/journal.pone.0094646 (PMC3983222; doi:10.1371/journal.pone.0094646)

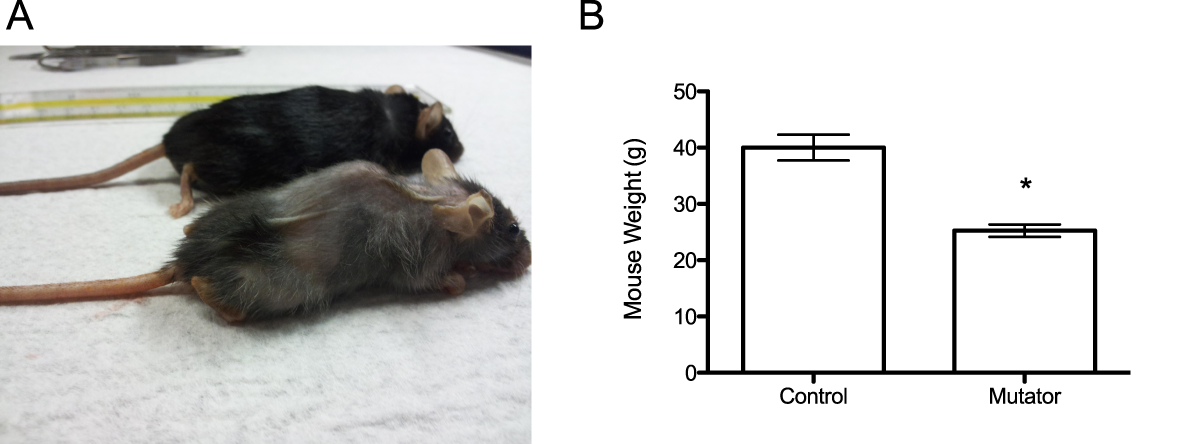

Supplement: Figure S1 — Polg mutator mice used in this study. A) Two male one year old littermates used in this study (WT/WT in background and Polg mutator in foreground). All of the mutator mice developed the characteristic progeroid phenotype. B) Mutator mice were significantly smaller than the controls used in the study at one year of age (control n = 4 (3 WT/WT and 1 WT/D257A) and mutator n = 7, * indicates t test p<0.05). (TIF) [file pone.0094646.s001.tif]
